# Pseudomyxoma Peritonei: An Incidental Diagnosis With Atypical Intraoperative Findings Discovered During a Laparoscopic Cholecystectomy

**DOI:** 10.7759/cureus.63426

**Published:** 2024-06-28

**Authors:** Michael W McGehee, Thomas V Thomas, Christopher J de la Houssaye

**Affiliations:** 1 Surgery, Edward Via College of Osteopathic Medicine, Blacksburg, USA; 2 Surgery, Holston Medical Group General Surgery, Kingsport, USA; 3 Radiology, Mountain Empire Radiology, Johnson City, USA

**Keywords:** general surgery, appendix tumours, atypical presentation, incidental cancer, indolent entity, intra-abdominal neoplasms, mucocele of the appendix, mucinous ascites, pseudomyxoma peritonei

## Abstract

Although pseudomyxoma peritonei (PMP) classically presents with profuse mucinous ascites within the peritoneal cavity, the physical manifestations of this disease exist on a spectrum, with the possibility of milder forms that lack typical findings. The authors report an indolent case of PMP diagnosed incidentally during workup and treatment for chronic cholecystitis in a 43-year-old male. This presentation of PMP was atypical due to a lack of discernible symptoms as well as uncharacteristic intraoperative findings consisting of numerous omental and pelvic adhesions with only sparse mucinous deposits. This case contributes to the growing understanding of PMP by exploring an uncharacteristic presentation of the disease with the hope that it may assist clinicians in diagnosing those cases of PMP that are more indolent and insidious in nature.

## Introduction

Pseudomyxoma peritonei (PMP) is a rare, chronic, and diagnostically challenging condition defined by intra-abdominal gelatinous ascites with mucinous peritoneal and omental implants [1]. The incidence of PMP is approximately one to three per million per year and is typically diagnosed in the sixth decade of life [2]. Pseudomyxoma peritonei most commonly originates from a mucinous tumor of the appendix. However, on rare occasions, PMP has been described as originating from other abdominal organs, including the stomach, pancreas, small intestine, and ovary [3]. The pathophysiology of PMP involves the continuous production of mucin by neoplastic epithelial cells. The mucin accumulates to form a mucocele, which eventually ruptures, allowing the free mucinous epithelial cells to implant on the peritoneum and omentum. These implanted tumor cells continue to proliferate and produce mucin, eventually leading to diffuse gelatinous ascites and associated complications secondary to increased intra-abdominal pressure [4]. There is no widely accepted standardized treatment regimen, but current recommendations suggest moving away from traditional debulking surgery and instead incorporating cytoreduction surgery to eliminate all visible lesions along with hyperthermic intraperitoneal chemotherapy to treat any remaining macroscopic or microscopic residues [5]. The five-year disease-specific survival probability following treatment with this regimen is approximately 60% [6]. This patient’s case of PMP was diagnosed incidentally while performing a laparoscopic cholecystectomy for treatment of the patient’s chronic cholecystitis and was atypical given the asymptomatic clinical presentation and relatively mild intraoperative findings.

## Case presentation

A 43-year-old Caucasian male presented to the emergency department with a chief complaint of sudden-onset, upper abdominal pain lasting for four hours. The patient also complained of nausea, vomiting, and mild shortness of breath. He denied having fatigue, changes in bowel movements, changes in skin color, or gastrointestinal bleeding. His medical history included an incarcerated umbilical hernia and a right inguinal hernia with subsequent surgical repairs. He had no pertinent family history. His social history included the use of tobacco chewing products and moderate alcohol use, but no smoking or illicit drug use.

**Figure 1:**
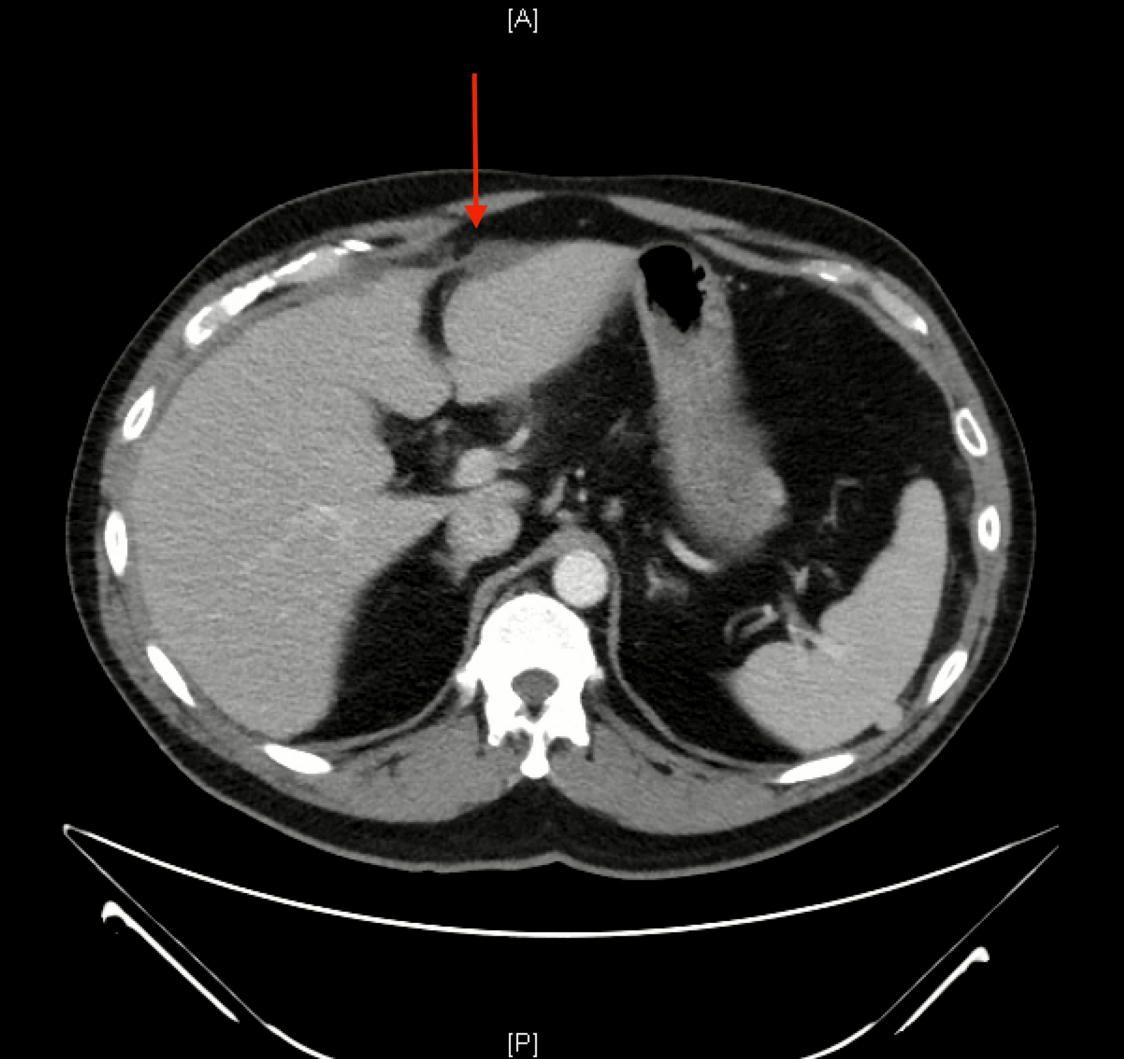
Axial CT scan of the liver The red arrow shows intraperitoneal free fluid anterior to the liver. A: anterior; P: posterior

**Figure 2:**
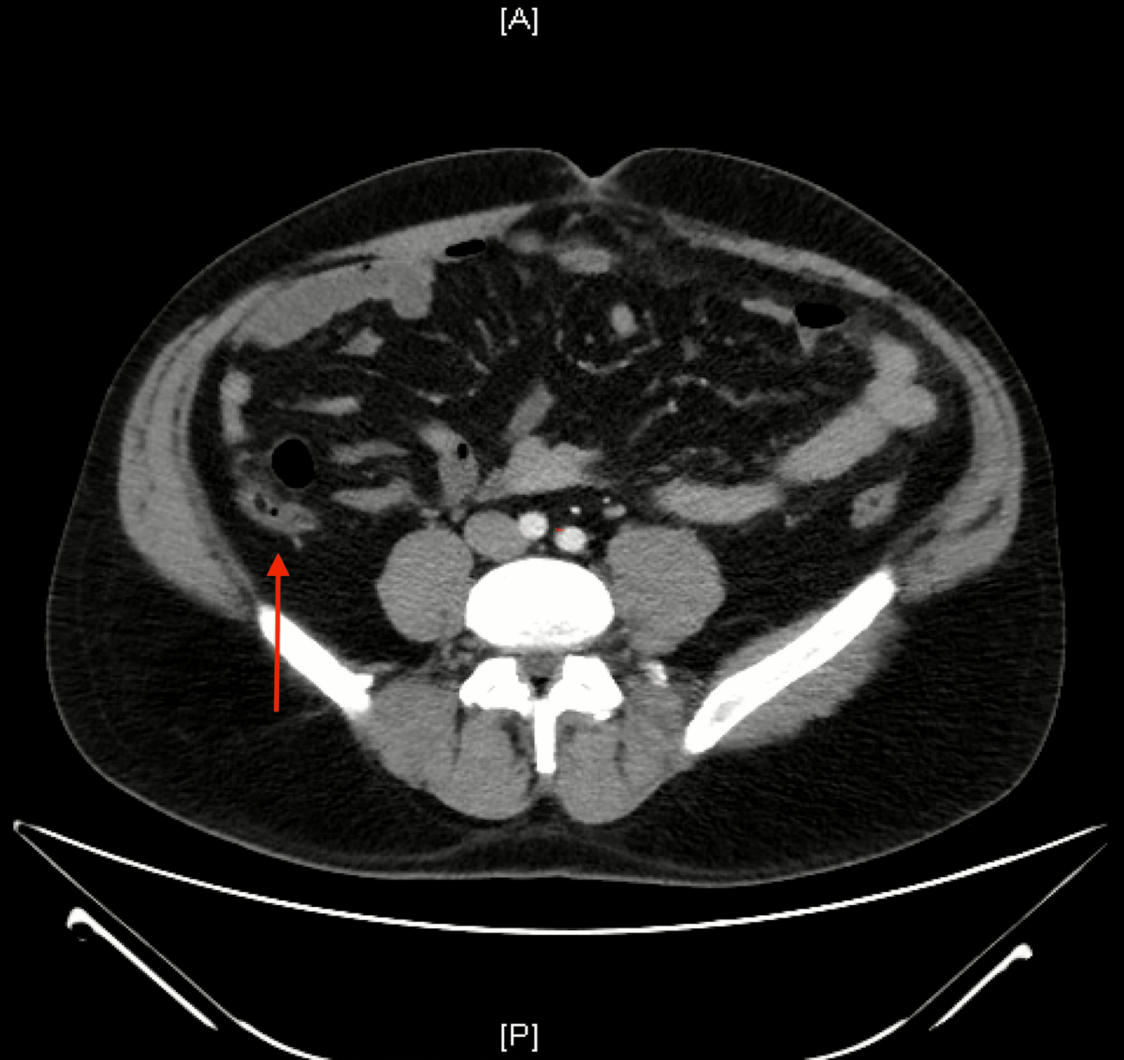
Axial CT scan of the lower abdomen The red arrow shows the enlarged appendix, which is distended by fluid. A: anterior; P: posterior

**Figure 3:**
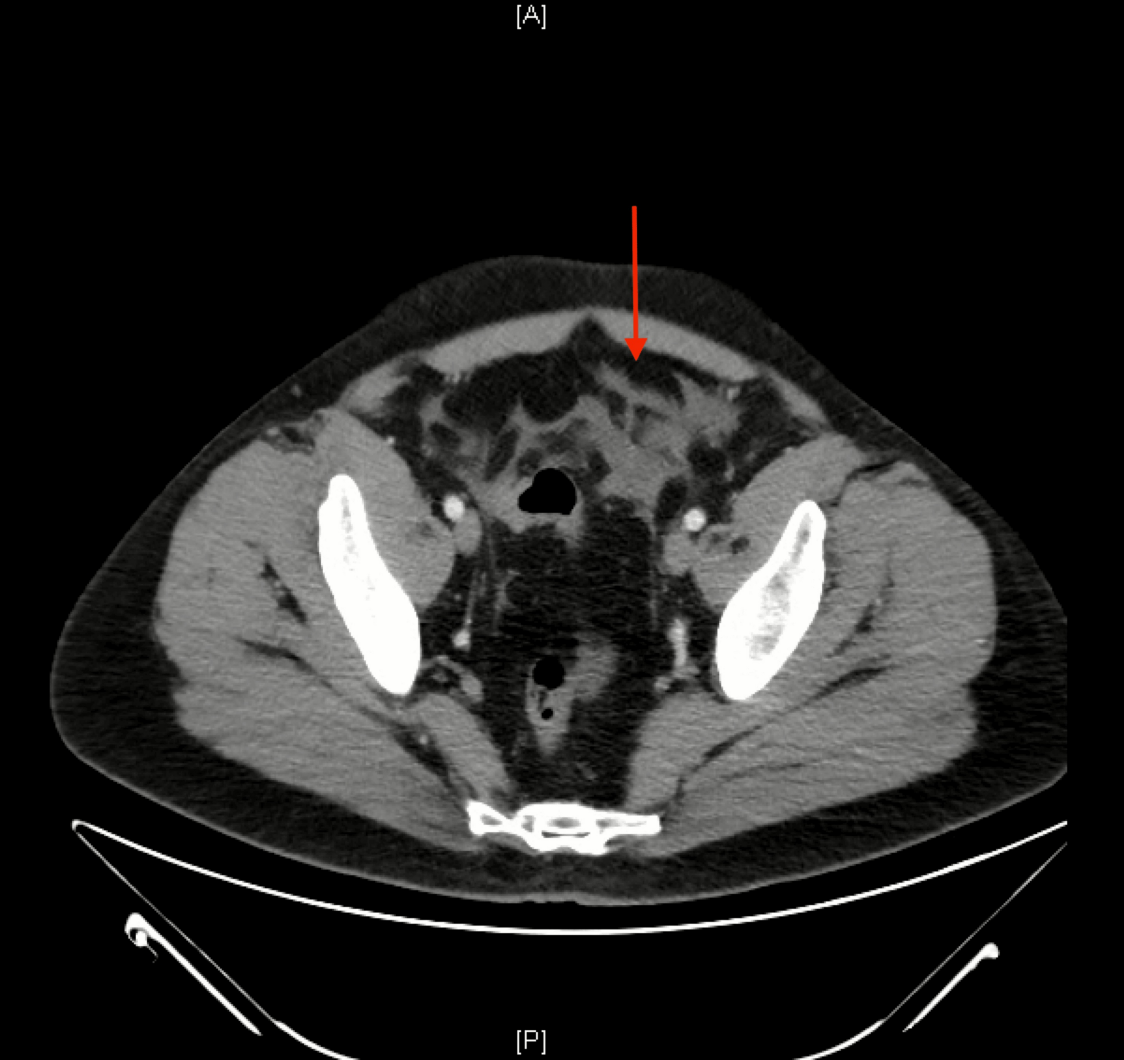
Axial CT scan of the pelvis The red arrow shows fat stranding and free fluid within the pelvic cavity. A: anterior; P: posterior

Upon review of objective data, the patient’s vitals were all within normal limits. His liver function tests and white blood cell count were also within normal limits. Upon physical examination, the patient was ill-appearing and exhibited tenderness to palpation in the epigastric area, but he did not have abdominal rigidity or distension, rebound tenderness, or abnormal bowel sounds. Both Murphy’s sign and McBurney’s sign were negative. A computed tomography (CT) scan of the chest, abdomen, and pelvis with contrast was ordered. There was a small amount of free fluid distributed throughout the peritoneal cavity (Figure 1). The gallbladder appeared normal in caliber, with no stones or dilation of the bile ducts. There was mild fat stranding along the gallbladder margin, although this was deemed nonspecific in the setting of free fluid. The appendix appeared prominent with mild fluid distension (Figure 2). However, it was noted that the appendix was poorly evaluated due to the distal aspect abutting loops of the bowel, which prevented a clear definition, as well as the presence of free fluid, which confounded the evaluation of possible inflammatory changes. Fat stranding and free fluid were also seen in the pelvic cavity (Figure 3).

The patient was treated with pantoprazole, famotidine, and ondansetron and experienced dramatic improvement in symptoms. Given the equivocal CT scan findings at the level of the appendix, the possibility of a repeat CT scan with oral contrast was discussed. However, follow-up CT imaging was deferred at that time in order to avoid additional radiation exposure. The patient was diagnosed with acute, unspecified gastritis by the emergency medicine physician and was discharged with prescriptions for similar medications. He was instructed to return for a repeat evaluation in the event that he experienced worsening symptoms.

Approximately six weeks later, the patient returned with a complaint of several episodes of right upper quadrant pain with associated nausea and vomiting. He noted that consuming greasy food is what precipitated these episodes. Upon review of objective data, the patient’s vital signs and lab values were within normal limits, and his physical examination was positive only for mild tenderness in the epigastrium. An abdominal ultrasound was performed, which showed the presence of multiple medium-sized gallstones. The patient was diagnosed with chronic cholecystitis and was recommended to undergo definitive treatment with a laparoscopic cholecystectomy. It was also recommended that the patient undergo a repeat CT scan to reassess the enlarged, fluid-filled appendix that was noted on the prior scan and evaluate for inflammatory changes and the presence of a possible appendiceal mucocele.

One month later, a CT scan of the abdomen and pelvis without contrast was performed. There was no significant radiological evidence of periappendiceal inflammation. There was once again a small amount of free fluid within the anterior pelvis adjacent to the sigmoid colon, which was similar in volume to what was seen on the initial CT scan. Finally, it was noted that there were new, worsening mesenteric nodules. A follow-up CT scan was scheduled for three months later, but the patient reported experiencing an improvement in symptoms and elected to cancel the scan and defer surgery until the symptoms became intolerable. 

Approximately nine months later, after experiencing worsening biliary symptoms, the patient presented to the surgery center to undergo an elective laparoscopic cholecystectomy for definitive treatment of his chronic cholecystitis. Upon obtaining laparoscopic camera access, it was discovered that the patient had extensive omental adhesions throughout the upper abdomen, extending inferiorly to the level of his previous umbilical hernia repair. Diffuse Fitz-Hugh-Curtis-type adhesions were present anterior to the liver. There were also extensive adhesions within the pelvic cavity as well as small, scattered mucin deposits. No free fluid was detected within the abdomen or pelvis. After lysing adhesions in the right upper quadrant, the gallbladder was removed without any complications. A small amount of mucus was suctioned out of the peritoneal cavity and sent for cytology. In addition, several small pieces of omentum and mucus were removed for pathological evaluation. There were no other obvious signs of malignancy. 

Upon examination by a pathologist, the samples from the pelvic adhesions were described as fibroconnective and fatty tissue with abundant dissecting acellular mucin pools. This raised the possibility of a ruptured mucous cyst, but also the possibility of a gastrointestinal tract neoplasm that had spread within the abdomen, likely of appendiceal origin. Immunostaining showed only rare cytokeratin 20 (CK20) positive cells, which were negative for CK7 and caudal type homeobox 2 (CDX2), suggesting the absence of an epithelial component and thus making the diagnosis of adenocarcinoma unlikely. Although there was no definitive evidence of a neoplastic process in the samples, a thorough clinical-radiographic investigation was recommended by the pathologist. Following the results of the pathology report, the patient was referred to an academic center with extensive experience in peritoneal neoplasms for further evaluation and treatment. 

## Discussion

After a retrospective review of this patient’s disease course, it was suspected that the patient had a small distal appendiceal mucocele, which ruptured sometime before the first CT scan was performed. The first CT scan, taken during the patient’s initial visit to the emergency department, showed an enlarged, fluid-filled appendix with a small amount of free fluid in the peritoneal cavity. The follow-up CT scan performed two and a half months later, showed a normal caliber appendix but increased nodules in the mesentery. This suggests that prior to the patient’s first visit, the appendiceal mucocele ruptured, which seeded the peritoneal cavity with mucinous tumor cells, resulting in a subclinical case of PMP. When compared to other cases of PMP described in the literature, this case is unique both in its clinical presentation and intraoperative findings.

From a clinical standpoint, this case of PMP was very indolent and unsuspected in nature. Appendiceal mucoceles are often asymptomatic and are generally found incidentally, which was the case with this patient [7]. However, once the mucocele ruptures, there are often acute symptoms such as right lower quadrant pain mimicking appendicitis, pelvic pain, or peritonitis [8]. On the contrary, this patient never experienced symptoms in the lower abdominal quadrants or pelvis; the only pain that he experienced was in the epigastrium and right upper quadrant, which were likely manifestations of his chronic cholecystitis. Furthermore, as PMP progresses into a chronic disease, symptoms relating to the characteristic mucinous ascites typically develop, which include abdominal distension, pain, changes in bowel habits, and bowel obstruction with associated fatigue and weight loss [9]. Again, this patient never experienced any of these ascites-related symptoms, but that is likely because he did not present with the full extent of physical manifestations that are usually observed during surgical exploration.

Typically, PMP is characterized by diffuse, profuse mucinous ascites. Mucin deposits tend to accumulate in predictable anatomical areas within the peritoneum where ascitic fluid pools due to the effect of gravity and is subsequently reabsorbed, known as the redistribution phenomenon. These areas include the greater omentum, the underside of the right hemidiaphragm, the right retrohepatic space, the left paracolic gutter, the ligament of Treitz, and the pelvis [10]. However, the operative findings in this patient’s case were less impressive compared to the extensive gelatinous ascites typically associated with PMP. Although there were numerous adhesions involving the omentum and pelvis, the mucin deposits were not extensive. The deposits were sparsely scattered throughout the peritoneal cavity and did not form the large confluent mucin pools typical of the disease.

## Conclusions

This case presents a unique and atypical manifestation of PMP that poses diagnostic challenges due to its indolent nature and largely asymptomatic presentation. While most cases of PMP present with distinct operative findings and discernible symptoms secondary to the accumulation of mucinous ascites, this patient had no overt symptoms related to the disease and had relatively mild operative findings consisting of omental and pelvic adhesions with sparse mucinous deposits. The contrast between this case and established PMP patterns underscores the importance of a thorough clinical-radiographic evaluation and collaboration with specialized centers for accurate diagnosis and management. It is important for physicians to consider PMP in the differential diagnosis of an asymptomatic patient with radiographic evidence of a fluid-filled appendix with subsequent peritoneal and pelvic abnormalities. This case contributes to the expanding knowledge of PMP and its variable presentations, emphasizing the need for vigilance and further research in understanding this rare and complex condition.
